# Micro-RNA Expression and Function in Lymphomas

**DOI:** 10.1155/2011/347137

**Published:** 2011-03-22

**Authors:** Sukhinder K. Sandhu, Carlo M. Croce, Ramiro Garzon

**Affiliations:** ^1^Department of Molecular Virology, Immunology and Medical Genetics, Molecular and Cellular Developmental Biology Program, The Ohio State University Medical Center, Columbus, OH 43210, USA; ^2^Department of Molecular Virology, Immunology and Medical Genetics and Comprehensive Cancer Center, The Ohio State University, Columbus, OH 43210, USA; ^3^Division of Hematology, Department of Internal Medicine and Comprehensive Cancer Center, The Ohio State University Medical Center, Biomedical Research Tower, Room 1084, 460 West 12th Avenue, Columbus, OH 43210, USA

## Abstract

The recent discovery of microRNAs (miRNAs) has introduced a new layer of complexity to the process of gene regulation. MiRNAs are essential for cellular function, and their dysregulation often results in disease. Study of miRNA expression and function in animal models and human lymphomas has improved our knowledge of the pathogenesis of this heterogeneous disease. In this paper, we attempt to describe the expression of miRNAs and their function in lymphomas and discuss potential miRNA-based therapies in the diagnosis and treatment of lymphomas.

## 1. Introduction

Recently, a class of noncoding RNAs called miRNAs has emerged as critical gene regulators in cell growth, disease, and development. MiRNAs are 18–24 nucleotide long noncoding RNAs, which regulate gene expression by pairing with 3′ untranslated region (UTR) of target mRNA and inhibiting protein translation and/or inducing mRNA degradation. MiRNAs modulate critical cell processes including cell growth, development, and differentiation. MiRNAs constitute approximately 1–3% of the genome and are predicted to regulate 30% of human genes. Currently, there are 940 miRNAs identified in humans and 590 in mice based on evolutionary conservation (miRBase 15, http://www.mirbase.org/). MiRNAs are transcribed by RNA polymerase II as long primary transcripts (pri-miRNA), which are processed into ~70 nucleotide long precursor miRNAs (pre-miR) by an RNAse-III-like enzyme, Drosha, together with DGCR8 (DiGeorge syndrome critical region gene 8), an RNA binding protein in the nucleus [1, 2]. Export of pre-miR to cytoplasm is mediated by Exportin-5 via GTP-dependent export, where it is further cleaved by another RNAse-III enzyme, called Dicer, into a mature dsRNA duplex. After strand-selection mature miRNA is assembled into the RNA-induced silencing complex (RISC), it ultimately performs regulatory function [3].

MiRNA-mRNA interactions are characterized by perfect or nearly perfect Watson-Crick base pairing involving 5′ miRNA seed region (typically bases 2–8) that binds the target mRNA [4]. A single miRNA is predicted to target about 300 mRNAs just as a single mRNA can be targeted by multiple miRNAs. mRNA targets for specific miRNA can be predicted by bioinformatics' algorithms (Targetscan, Pictar, Miranda), but validation must be achieved through luciferase reporter assays, quantitative real-time PCR, and immunoblotting.

In addition to the canonical mechanisms of miRNA gene regulation through 3′ UTR interactions, other “noncanonical” miRNA-mediated mechanisms of mRNA expression modulation are emerging (reviewed in Garzon et al. [5]). Some miRNAs have been shown to bind to the open reading frame or to the 5′ UTR of the target genes and, in some cases, activate rather than inhibit gene expression [5]. Our group has recently reported that miRNAs exhibit decoy activity and bind to ribonucleoproteins in a seed sequence and an RISC-independent manner and interfere with their RNA binding functions [5]. Few studies have reported that miRNAs can also regulate gene expression at the transcriptional level by binding directly to the DNA [5]. Overall, these data show the complexity and widespread regulation of gene expression by miRNAs that should be taken into consideration when developing miRNA-based therapies.

Analysis of miRNA localization in the genome by Rodriguez et al. [6] yielded useful information into their mode of transcription. Based on their location, miRNAs can be intergenic, intronic, or exonic and can be transcribed as a single miRNA from its own promoter, (monocistronic) or several miRNAs as a cluster from a shared promoter, (polycistronic). Intergenic miRNAs are found in between genes in distinct transcription units. miRNAs can be intronic of coding or noncoding genes, where they may be transcribed from the same promoter as the host gene. The exonic miRNAs are rare and are mainly found in exons of coding or noncoding genes. A study from our lab showed that in mouse, miRNA genes are frequently located near cancer susceptibility loci, which are often subjected to genomic alterations leading to activation by translocations or amplifications, or loss of function due to deletions, insertions, or mutations [7].

In addition to the above structural genetic alterations, various epigenetic events can also lead to miRNA expression deregulation. miRNA promoter hypermethylation and/or histone hypoacetylation has been described in solid tumors and hematological malignancies [8–10]. Furthermore, treatment with methylating agents or histone deacetylases inhibitor drugs leads to activation of tumor suppressor miRNAs like *miR-127* which targets proto-oncogene BCL6 in human bladder cancer cells [8]. Aberrant miRNA expression may also result from downstream miRNA processing. For example, short hairpin-mediated silencing of Dicer and Drosha (RNAses involved in miRNA processing) can lead to global repression of miRNA expression promoting cellular transformation and tumorigenesis in vivo [11]. Finally, miRNA activation can also result from increased transcription from their respective host genes due to aberrant transcription factor activity. For example, activation of *miR-34a*, *miR-34b*, and *miR-34c* family of miRNAs by tumor suppressor p53 may in part contribute to its tumor suppression activity [12, 13].

Deregulated miRNA expression is reported in various human diseases including lymphomas, suggesting an important role in their pathogenesis. Animal studies have established that a subset of miRNAs play a role in the initiation and progression of lymphomas. The purpose of this paper is to synthesize the current work on miRNA profiling of lymphomas in light of developing miRNA therapeutic strategies. The potential implications for prognosis and possible uses in diagnosis and treatment are also discussed. Two of the most notorious miRNAs upregulated in various lymphomas, *miR-155* and *miR-17~92*, are discussed in detail, since these miRNAs are among the best studied owing to the availability of both gain- and loss-of-function mouse models.

### 1.1. miRNAs in Lymphomas: miRNA-155

The original *miR-155* was encoded from a common retroviral integration site in avian leukosis virus-(ALV-) induced B-cell lymphomas, called *bic* for B-cell integration cluster and later shown to cooperate with c-MYC to induce lymphomagenesis [14]. Earlier studies showed *miR-155* was upregulated in pediatric Burkitt's lymphoma [15], aggressive activated B-cell-like (ABC) subtype of DLBCL [16], primary mediastinal B-cell lymphoma (PMBL) and Hodgkin's lymphoma [17], and CLL [18]. Since *miR-155* is frequently upregulated in B-cell lymphoproliferative disorders, Carlo Croce's group developed transgenic mice that overexpress *miR-155* in B cells under the E*μ* promoter. These mice were viable and fertile but developed a polyclonal lymphoproliferative disorder followed by pre-B-leukemia/lymphoma at a young age. This was the first evidence that a single miRNA deregulation could cause cancer [19]. The oncogenic nature of this miRNA was reinforced by identification of *miR-155 *orthologue (miR-K12-11) in the Kaposi sarcoma-associated herpesvirus (KSHV) associated with B-cell tumors [20], and recently from Marek's disease virus of chickens [21]. The mechanisms involved in *miR-155* induced lymphomagenesis are still under extensive investigation. However, two independent studies recently indicate that *miR-155* represses SH2-domain containing inositol-5-phosphatase-1 (SHIP-1), which is a critical phosphatase that negatively downmodulates AKT pathway and has functions during normal B-cell development [22]. Thus, sustained overexpression of *miR-155* in B cells unblocks AKT activity, inducing B-cell proliferation. Similar results were reported in the myeloid cells, resulting from myeloproliferation in mice transplanted with *miR-155* transduced murine bone marrow HSCs [23]. Hematopoietic reconstitution assays showed extensive myeloproliferation with associated splenomegaly and morphological dysplastic changes. In addition to SHIP-1, C/EBP*β*, PU.1, and CSFR are also validated *miR-155* targets. Physiologically, *miR-155* is upregulated during B-cell activation in the germinal centers upon antigen stimulation and hence plays a role in antibody class switching and plasma cell formation, both of which are impaired in the *miR-155*-deficient mice [24, 25]. In another study, *miR-155*/BIC was transcriptionally regulated during normal B cell receptor activation through the AP-1 transcription factor and extracellular signaling-regulated kinase (ERK) and c-Jun N-terminal kinase JNK pathways [26]. Above findings show critical role of *miR-155 *in immune cell development, differentiation, function, and immune response regulation, dysregulation of which can lead to malignancies like lymphoma.

### 1.2. miRNAs in Lymphomas: miR-17~92

Another miRNA which is reported to have a major role in lymphomagenesis is the *miR-17~92* polycistron located in 13q31-32, a region commonly amplified in B-cell lymphomas and upregulated in 65% of the B-cell lymphoma patients [27, 28]. The *miR-17~92* cluster consists of *miR-17*, *18a*, *19a*, *19b-1*, *20a*, and *92a-1* and is encoded from the last exon of noncoding RNA called C13orf25. The cluster has two paralogs in the genome, *miR-106a~363* on chromosome X in mice and humans consisting of six miRNAs, and *miR-106b~25* on chromosome 5 in mice (chromosome 7 in humans) consisting of three miRNAs encoded from the 13th intron of the DNA-replication gene Mcm7 [29].

Gain and loss-of-function studies of *miR-17~92* polycistron have provided an important insight into its mechanism of action and its targets. He et al. demonstrated that virus-mediated overexpression of *miR-17~92* in lymphocytes of e*μ*-MYC (B-cell) transgenic mice accelerated tumor development [28]. O'Donnell et al. simultaneously reported that MYC binds and activates expression of the *miR-17~92* cluster [30]. Two members of the polycistron, *miR-17-5p* and *miR-20a*, downregulate E2F1, which is a direct target of MYC that promotes cell cycle progression. More recently, Xiao and colleagues [31] reported that mice with sustained expression of *miR-17~92* in lymphocytes exhibit a lymphoproliferative disorder, autoimmunity, and premature death. These mice have decreased levels of the proapoptotic BIM and the tumor suppressor PTEN. Both genes were further confirmed as targets of the *miR-17~92* polycistron members [31]. Ventura et al. [29] confirmed these findings and showed that targeted deletion of *miR-17~92* polycistron (but not its paralogs) in mice is embryonically lethal and critical for lung and B-cell development. In particular, these mice exhibited a block in B-cell differentiation at the pro-B to pre-B transition caused by high levels of the proapoptotic protein BIM. These experiments suggest that the *miR-17~92* cluster acts specifically during the transition from pro-B to pre-B lymphocyte development, enhancing the survival of the B-cells at this stage by targeting the proapoptotic BIM. Further dissection of the genetic complexity of the cluster was demonstrated by generating conditional knockout alleles of the four seed regions represented in the cluster: *miR-17*, *miR-20a*; *miR-18a*; *miR-19a*, *miR-19b-1*; *miR-92-1* [32]. Mu and colleagues [32] found that deletion of the whole *miR-17~92* cluster slows c-Myc-induced oncogenesis. This phenotype was rescued by reintroduction of the full cluster, but not by the cluster lacking *miR-19a* and *miR-19b*, thereby suggesting *miR-19* as the most important miRNA of the cluster. Using a different approach, Olive and colleagues [33] overexpressed individual miRNAs in the E*μ*-MYC mice model. They found that overexpression of the whole cluster, without *miR-92*, but not the *miR-19a* or *miR-19b*, promotes oncogenesis. In summary, both studies indicate that *miR-19* is critical for the oncogenic activities of this cluster. Recently, *miR-17~92* was also shown to downregulate TGF*β* signaling pathway leading to clusterin downregulation and hence stimulating angiogenesis and tumor cell growth in glioblastomas [34].

## 2. Micro-RNA Expression Profiling Studies

Strong experimental data supported a critical role for miRNAs in lymphomagenesis; several groups performed systematic miRNA profiling in lymphoma patient samples using a variety of miRNA expression platforms. We will summarize the miRNA expression data in lymphoma according to the two main categories: Hodgkin lymphoma (HL) and non-Hodgkin lymphoma (NHL) along with its subcategories.

### 2.1. Hodgkin Lymphoma

Hodgkin lymphoma (HL) is one of the most curable forms of cancer, common in younger people and is characterized by the presence of Hodgkin/Reed-Sternberg (HRS) cells. HL is derived from mature B cells and subdivided into classical Hodgkin lymphoma (cHL) and nodular lymphocyte predominant Hodgkin lymphoma (NLPHL). HL is unique among B-cell lymphomas because of the rarity of the lymphoma cells, called HRS cells in cHL and lymphocyte predominant (LP) cells in NLPHL, which makes up to 0.1% to 10% of the cells in the affected tissues [35]. The HRS cells are characterized by the loss of B-cell specific gene expression, and these cells show constitutive NF-*κ*B activity, which may be responsible for their enhanced survival [35]. The most common form of HL is the classical Hodgkin lymphoma (cHL) and represents scarcity of tumor cells in lymphoma biopsies, which limits the genetic analysis of HRS cells [36]. Comparison of miRNA expression of microdissected HRS cells from cHL patients to CD77+ GC B cells showed three downregulated miRNAs, namely, *miR-520a*, *miR- 200a*, and *miR-614* and twelve upregulated miRNAs, namely, *miR-20a*, *miR-21*, *miR-9*, *miR-155*, *miR-16*, *miR-140*, *miR-18a*, *miR-30b*, *miR-30a*-*5p*, *miR-196a*, *miR-374*, and *miR-186 * [36]. The functional implications of these results are still to be investigated, but some of the most common predicted targets of the upregulated miRNAs are members of the SOCS (Suppressor of Cytokine signaling) family which may contribute to activated JAK/STAT signaling in the HRS cells due to miRNA-mediated SOCS inactivation. Studies from HL cell lines showed *miR-9* to be one of the ten most significantly upregulated miRNAs and target B-lymphocyte-induced maturity protein 1 (Blimp1 or PRDM1) which plays an important role in plasma cell differentiation [37].

miRNA profiling study from 49 cHL patient samples and 10 reactive lymph nodes (RLNs) using real time PCR revealed three distinct disease groups: nodular sclerosis cHL, mixed cellularity cHL, and RLNs, signifying the role of miRNAs in predicting prognosis [38]. HL-specific miRNA signature from HL cell lines includes *miR-17~92*, *miR-16*, *miR-21*, *miR-24*, and *miR-155* being upregulated and *miR-150* as the only downregulated miRNA [39]. About 40–60% of HL patients have EBV (Epstein Bar Virus) associated with the malignant cells, but precise role of EBV in cHL pathogenesis is unknown [38]. Complex interactions among viral and host miRNAs can contribute to lymphomagenesis by targeting multiple pathways. EBV was shown to influence miRNA in cHL with 10 host miRNAs differentially expressed in EBV+ and EBV− cases [38]. It is hypothesized that EBV can lead to NF-*κ*B activation responsible for survival of HRS cell [40]. Previously, EBV has been shown to transactivate *miR-155* through NF-*κ*B activation and hence may be an important pathogenesis event in cHL [41]. The miRNA that seems critical for the survival advantage of cHL cells is *miR-21* which can indirectly upregulate antiapoptotic genes [38].

The differences in miRNA expression profile of HRS cell by Van Vlierberghe et al. [36] and primary cHL patient samples and HL cell lines by Gibcus et al. [39] and Navarro et al. [38] highlight the disparity between the expression profile of the tumorous HRS cell and the nonneoplastic surrounding cells. It also signifies the intricate interaction between the HRS cell and the surrounding microenvironment, which is important in understanding the disease biology.

### 2.2. Non-Hodgkin Lymphoma

NHL is an increasingly common cancer with over 60,000 cases every year in the US and includes more than 20 lymphoproliferative malignant diseases that originate from T and B lymphocytes and which can be aggressive (fast growing) or indolent (slow growing). B-cell NHLs include diffuse large B-cell lymphoma, mantle cell lymphoma, Burkitt's lymphoma, follicular lymphoma, and rare lymphomas. Still other categories include chronic lymphocytic leukemia/lymphoma, immunoblastic large cell lymphoma, and precursor B-lymphoblastic lymphoma. T-cell NHLs include mycosis fungoides, anaplastic large cell lymphoma, and precursor T-lymphoblastic lymphoma. Based on the cell of origin and degree of differentiation, various subcategories of non-Hodgkin lymphoma have been established according to the 2008 classification of tumors of hematopoietic and lymphoid tissues by World Health Organization. miRNA profiling studies available in the literature for selected are discussed.

#### 2.2.1. Diffuse Large B-Cell Lymphoma (DLBCL)

DLBCL is the most common type of NHL that represents over one-third of new diagnoses (~20,000 new cases per year) [42]. Gene expression profiling has been used to classify it into three major histologically indistinguishable subgroups: germinal center B-cell-like DLBCL (GCB-DLBCL), activated B-cell-like (ABC-DLBCL), and primary mediastinal DLBCL (PMBCL) [43]. This subclassification is also achieved by immunostaining for CD10, BCL6, and MUM-1 [44] and has been incorporated in the 2008 World Health Organization classification of lymphoid tumors [45]. The three subgroups have different clinical outcomes with respect to the treatment regimen (CHOP and R-CHOP) with a 5-year survival rates of 59%, 30%, and 64% in patients with GCB-DLBCL, ABC-DLBCL, and PMBCL, respectively [46]. From the cytogenetics point of view, GCB lymphomas are characterized by t(14;18) translocation, deletion of tumor suppressor PTEN, and amplification of micro-RNA cluster *miR-17~92* and p53 mutations [47]. ABC lymphomas are known for antiapoptotic BCL2 amplification, and majority of them have deletion of tumor suppressor loci like INK4-ARF which encodes p16, an inhibitor of senescence and p14ARF, an inhibitor of p53 activation [47]. Loss of these tumor suppressors contributes to poor prognosis and resistance to chemotherapy in ABC type of lymphomas. PMBL (Primary mediastinal B-cell lymphoma) is characterized by amplification of 9p24 region of chromosome 9 in half of the patients with this subtype and also in Hodgkin lymphoma. This region encompasses many megabases of DNA, but one major candidate is JAK2, a tyrosine kinase that phosphorylates and activates the transcription factor STAT6. Furthermore, SOCS1 (suppressor of cytokine signaling), target of *miR-155* is regularly deleted in PMBL and Hodgkin's lymphoma.

Recently, miRNA expression profiling studies have been shown to successfully classify DLBCL into it subcategories as defined by mRNA and immunohistochemistry. A 9-miRNA signature (*hsa-miR-146b-5p*, *hsa-miR-146a*, *hsa-miR-21*, *hsa-miR-155*, *hsa-miR-500*, *hsa-miR-222*, *hsa-miR-363*, *hsa-miR-574-3p*, and *hsa-miR-574-5p*) from study by Malumbres et al. [48] can precisely differentiate the DLBCL into ABC or GCB subtypes, and expression of some of these miRNAs correlated with clinical outcome. It was also shown that during normal B-cell development, *miR-125b* is enriched in GC B cells and keeps IRF4 and PRDM1/Blimp1 down and *miR-223* is enriched in memory B cells where it targets and downregulates LMO2 [48]. The reciprocal relationship between miRNAs and their targets in these cells signifies the physiological relevance whereby downregulation of IRF4 and PRDM1 in GC B-cells is essential for progression of these cells to memory (post GC) B cells which downregulates LMO2 and upregulates IRF4 and PRDM1. Interestingly, they also came up with a 39 miRNA signature which can successfully differentiate between distinct B-cell subpopulations comprising the “cell-of-origin classifier.”

**Figure 1:**
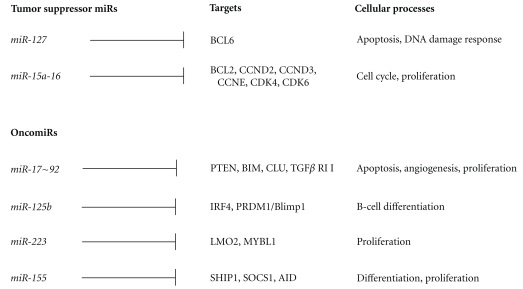
Tumor suppressor miRNAs and oncomiRs in DLBCL and their target genes implicated in various processes involved in malignant transformation.

Unique categorization of 86 DLBCLs was reported by genome wide miRNA expression profiling into three subtypes, namely, MiRNA Group/MG-A, B, and C [49]. The new classification is unrelated to already known GCB and non-GCB classifications. Distinctive miRNA signatures obtained using unsupervised hierarchical clustering could distinguish these three groups based on just 16 miRNAs with *miR-17~92* cluster members (*miR-17-5p*, *miR-17-3p*, *miR-18a, miR-19a, miR-20a, miR-20b*, and* miR-92*) and its paralog *miR-106a*, being the predominant one in addition to *miR-29a/c,miR-100*, miR-199a*, *miR-140*, *miR-630*, and *miR-16 * [49]. Furthermore, patients with the MG-A signature characterized by upregulation of the *miR-17~92* cluster and its paralog *miR-106a-363* of chromosome X had the worst overall survival. This miRNA signature-based classification of DLBCL highlights a significant role of MYC as, unlike the *miR-17~92*, which is activated by MYC [30], 95% of the miRNAs which are suppressed by MYC were significantly downregulated in the MG-A subgroup and overexpressed in the MG-C [49]. In addition, integration of copy number and expression data in these samples showed overexpression of *miR-100*, *miR-125b*-1, and *miR-130a* (on chromosome 11, and members of the MG-B cluster) as a consequence of chromosomal gain or amplification [49]. However, no copy number loss at chromosome 13q14 influenced the expression of *miR-15a/16-1*, loss of which is often attributed to common B-cell malignancies like CLL [49, 50]. This study also uncovered a novel set of miRNAs like *miR-222* and *let-7f* (associated with other malignancies), *miR-513* and *miR-223* (linked to immune regulation and related B-cell tumors), *miR-424 *(hematopoiesis), and *miR-188* and *miR-374 *(no known physiological or pathological functions) [49]. Interestingly, several of these miRNA loci map to chromosome X, but the fact that no candidate target genes in this region have been ascribed to any copy number changes in DLBCL leads to the significance of the role of miRNA deregulation in this disease [49]. It is worth mentioning that Li et al. [49] provide an important miRNA expression signature of DLBCL, linked to copy number changes targeting the miRNA genome, MYC activity, and is unrelated to the initially established cell-of-origin classification. miRNA signature associated with event-free and overall survival in DLBCL was reported to include: *miR-21*, *miR-127*, *miR-34a*, *miR-195*, and *miR-let7g *[51]. In addition, few lymphoma-specific miRNA signature included *miR-150*, *miR-17-5p*, *miR-145*, *miR-328*, and others [51]. One of the most commonly deregulated miRNAs in DLBCL is *miR-155*, and some of its validated targets, which have role in B-cell homeostasis, include PU.1, AID, and SOCS1. In another study *miR-155* overexpression was shown to make DLBCLs resistant to the growth-inhibitory effects of both TGF*β*1 and bone morphogenetic protein (BMP), via defective induction of p21 and impaired cell cycle arrest through targeting SMAD5 [52]. Genomic profiling of Richter's syndrome, which represents a transformation of chronic lymphocytic leukemia (CLL) to aggressive lymphoma and is mostly represented by DLBCL, with a postgerminal center phenotype, shows a 13q amplification which encodes the *miR-17~92* cluster which interacts with c-MYC [53]. Since DLBCL can be considered as a prototype of human lymphomas, role of miRNAs and their respective targets in lymphomagenesis is depicted in Figure 1.

#### 2.2.2. Mantle Cell Lymphoma (MCL)

Mantle cell lymphoma (MCL) is a subtype of NHL which usually arises due to malignant transformation of a B lymphocyte in the outer edge of a lymph node follicle, called the mantle zone and resulting in lymph node (LN) enlargement. MCL accounts for about 6% (~3500) of all new NHL cases in the United States each year and has the worst prognosis, with average age at diagnosis in mid-60s. MCL is characterized by overexpression of cyclin D1 (ccnd1), which is usually because of a reciprocal translocation, t(11;14)(q13;q32). Like other B-cell lymphomas, MCL is treated with a multidrug therapy involving Bortezomib or autologous stem cell transplant.

Several miRNAs have been implicated in MCL pathogenesis. miRNA expression profiling of B cells from MCL patients led to 15 up- and 18 downregulated miRNAs. *miR-29* family (*miR-29a*, *miR-29b*, and *miR-29c*) was among the major downregulated miRs in addition to *miR-142-3p/5p*, *miR-150*, and *miR-15a/b*, and associated with short overall survival [54]. Among the upregulated miRNAs were *miR-124a*, *miR-155*, *miR-328*, *miR-326*, *miR-302c*, *miR-345*, miR-373*, and *miR-210 * [54]. They also showed that *miR-29* targets CDK6, expression of which is a known prognostic and pathogenetic factor in MCL. Furthermore, downregulation of *miR-29* is in line with the ccnd1 overexpression and the consequent CDK4/CDK6 activation, which is the primary event in MCL pathogenesis. The patients with significantly downregulated *miR-29* had a shorter survival compared with those who expressed relatively high levels of *miR-29 * [54]. Loss of *miR-29a* and *miR-29c* also correlates with TCL1A overexpression in MCL, and direct regulation of it has been previously shown [55].

According to another study, *miR-31*, *miR-148a*, and *miR-27b* were among the downregulated, and *miR-617, miR-370*, and *miR-654* were among the upregulated miRNAs in 23 MCL cases as compared to 11 reactive lymphoid tissues [57]. One of the predicted targets of *miR-31* (most downregulated miRNA) is MAP3K14 (NIK), a gene essential for activation of the alternative NF-*κ*B pathway [57]. Interestingly, the miRNA clusters at locus 7q22 including *miR-106b, miR-93*, and *miR-25* (overlapping *miR-17~92* cluster) were also highly upregulated in MCL. Tumor suppressors targeted by this cluster include PTEN, and the proapoptotic BIM and *miR-106b* specifically promotes cell-cycle progression by targeting cyclin-dependent kinase inhibitors p21/cdkn1a. In addition, *miR-106b* overrides doxorubicin-induced DNA damage checkpoint [60]. *miR-20b*, another member of the oncomir-1 cluster, had a significant clinical prognostic value, loss of which was associated with better prognosis in MCL similar to several other diseases like gastric cancer, T-cell leukemia, and mammary tumors [57].

#### 2.2.3. Burkitt Lymphoma (BL)

According to WHO classification, Burkitt lymphoma (BL) is a rare, highly aggressive NHL composed of monomorphic medium-sized B cells with multiple nucleoli and numerous mitotic figures and is more common in children than in adults [61]. The tumor exhibits a starry-sky pattern because of the histiocytes with phagocytosis of apoptotic cellular debris [62]. BL has been referred to as “The Rosetta Stone of Cancer” because of the many Firsts: first human cancer shown to be associated with virus, chromosomal translocation leading to activation of an oncogene, and the first cancer to be successfully treated with chemotherapy alone [63].

Cytogenetically, almost all BL cases have a translocation involving the MYC gene at 8q24 with the immunoglobulin heavy chain gene (IGH) on 14q32 or less commonly with kappa light chain locus (IGK) at 2q11 or light chain locus (IGL) at 22q11 [26, 49] and the Epstein Bar Virus (EBV) associated with most aggressive cases. Both, classic and variant translocations involving MYC activation are associated with PVT1 oncogene, which encodes seven miRNAs including *hsa-miR-1204* responsible for c-myc activation [66]. Robertus et al. (2010) recently showed myc-related miRNA profile that can characterizes Burkitt lymphoma from other B-NHL (B-CLL, MCL & FL) [67]. Two of the miRNAs downregulated in BL cell lines, EBV transformed B-cell lines, CLL, and B cell lymphomas, are *miR-143* and *miR-145*, expression levels of which are inversely associated with cell proliferation [68]. Moreover, *miR-143* and *miR-145* are proposed to modulate MAPK signaling cascade via downregulating ERK5, and other members of the pathway to affect cell growth and survival [68]. Expression analysis to characterize various human B-cell stages and malignancies derived from them yielded nineteen miRNAs which characterized BL from other B-cell lymphomas (Table 2) [58].

#### 2.2.4. Follicular Lymphoma/Indolent Lymphoma

Indolent or slow growing NHL includes follicular lymphoma (FL), tumor of germinal center origin, which accounts for 20–30 percent of all NHL and usually takes several years to develop. It has the tendency to transform into DLBCL, with translocation t(14;18)(q23;q21) in 90% of cases and is associated with BCL2 activation which may lead to accumulation of GCB cells with prolonged lifespan. In addition, this transformation may lead to c-myc gene rearrangement, p53 or BCL6 mutation, or inactivation of tumor suppressors p15 or p16 [69]. Comparative miRNA expression analysis of 46 FL cases to lymph node samples was used to generate FL miRNA signature which included miRNAs which have role in hematopoiesis (*miR-150* and *miR-155*) or tumor development (*miR-210*, *miR-10a*, *miR-17-5p*, and *miR-145*) [51]. Few FL-specific miRNAs included miR-9/9*, *miR-301*, *miR-338*, and *miR-213 *[51]. 

## 3. Rare Lymphomas

Among the rare lymphomas are the peripheral T-cell lymphomas (PTCL) like enteropathy-associated T-cell lymphoma (EALT), gamma delta T-cell lymphoma, anaplastic large cell lymphoma (ALCL), primary cutaneous PTCL, and lymphocytic variant hypereosinophilic syndrome [59]. MiRNA expression profiling study of tumor cells (T-cell clones) of a rare, aggressive, primary cutaneous T-cell (CD4+) lymphoma called Sezary syndrome (SzS) showed that majority of deregulated miRNAs were downregulated including oncomir-1/*miR-17-5p *[59]. They further showed that ectopic expression of *miR-17-5p* increased apoptosis and decreased cell proliferation in SzS cells [59]. Furthermore, the four miRNA shaving a significant diagnostic potential (100% accuracy) for SzS included *miR-150*, *miR-191*, *miR-15a*, and *miR-16.* Other miRNAs which could also distinguish SzS samples from healthy controls with 96% of accuracy were *miR-223* and *miR-17-5p* [59]. Ballabio et al. [59] also showed that direct targeting of EVL which is the host gene of *miR-342*, by the upregulated miRNA, miR-199a* was responsible for its downregulation. EVL gene has been implicated in carciongenesis of colorectal cancers where it is silenced by CPG methylation. Out of the 114 differentially expressed miRNAs, the only 10 upregulated miRNAs in SzS samples were *miR-145*, *miR-574-5p*, *miR-200c*, miR-199a*, *miR-143*, *miR-214*, *miR-98*, *miR-518a*-*3p*, and *miR-7*. The aberrant expression of MYC in SzS was found to correlate with the set of miRNAs including *miR-30*, *miR-22*, *miR-26a*, *miR-29c*, *miR-30*, *miR-146a*, and *miR-150* which were downregulated.

Another miRNA profiling study of a premalignant T-cell clone in lymphocytic variant hypereosinophilic syndrome (L-HES) unraveled a set of downregulated miRNAs which are predicted to target the three upregulated mRNAs: RBBP8, CLU, and MAP3K8. The retinoblastoma binding protein 8 gene (RBBP8) is a predicted target of *miR-31*, *miR-126*, *miR-130a*, and *miR-335* and is a putative tumor suppressor. On the other hand, Clusterin (CLU) is a predicted target of *miR-99a*, *miR-100*, *miR-126*, and *miR-335* and is commonly associated with tumorigenesis and malignant progression in part through *TGF-miR-135a*, *miR-135b*, *miR-181a*, and *miR-181b * [65]. Another miRNA whose expression significantly decreases with the progression of L-HES to T lymphoma was *miR-125a*. Accordingly, its predicted target genes, PTPRN2 (member of the receptor-type protein tyrosine phosphatase family) and AHI1 (Abelson helper integration site 1), were significantly upregulated [65]. *miR-125b* (homolog of *miR-125a*) has been shown to target TNF*α* and decrease cell proliferation in mouse macrophage cells [70]. The exact role of *miR-125b* is complicated by the fact that it is predicted to target more tumor suppressors than oncogenes, and one of the most important predicted candidates is the antiapoptotic MCL1 (Myeloid cell leukemia 1), a gene frequently upregulated in CLL and myeloid leukemias. Further studies will help understand its role in immune cell development and lymphoma genesis. 

## 4. Potential for miRNAs in Prognosis and Outcome Prediction

miRNA expression profiling studies have been successfully used not only to differentiate normal from cancer tissues but also to classify tumor types and grades. In addition, miRNA expression profiling can also provide a unique miRNA expression signature, which could be related to response to therapy and survival through [38, 58]. Li et al. [49], Roehle et al. [51], and others have successfully used miRNA expression profiles to determine unique miRNA signatures, which can classify lymphomas into categories with distinct treatment response and outcome in the patients (Table 1). A recent report by Lawrie et al. [71] showed *miR-21* serum levels to be associated with relapse-free survival in patients with DLBCL. Similarly, serum levels of *miR-141* have been shown to distinguish between prostate cancer patients and healthy individuals [72]. In MCL, *miR-20b* expression is related to poor survival, and its lack of expression distinguished cases with a survival probability of 56% at 60 months [57]. One of the miRNAs consistently downregulated in most lymphomas is *miR-150*, owing to its important role as tumor suppressor as shown in a loss-of-function mouse model [73]. Mice lacking *miR-150* have increased expression of its target transcription factor, c-Myb oncogene, which plays an important role in lymphocyte development and maturation [73]. Therefore, miRNA expression profiling provides a useful tool for prognosis, diagnosis, and outcome prediction in lymphoma patients.

## 5. Therapeutic Potentials for miRNAs in Lymphomas

Since miRNA expression is deregulated in several lymphomas, and unique miRNA signatures have been identified for prognosis and response to therapy, they become quiet appealing as therapeutic targets. Common therapeutic strategies involve antisense-mediated inhibition of oncogenic miRNAs and miRNA mimetics or viral-vector encoded overexpression of tumor suppressor miRNAs. Some of the strategies in the former category involve oligonucleotides with chemical modifications, liposomes, polymers, hydrogels, and nanoparticles [5]. Synthetic anti-miRNA oligonucleotides (AMOs), which have 2-O-methyl modification, provide an effective inhibition of miRNAs in cell culture and xenograft mouse models but work only at high doses [74]. Targeting of *miR-21* by 2-O-methyl AMOs in glioblastoma and breast cancer has been achieved in vitro and xenograft mice model, respectively [75, 76]. An alternative to AMOs is locked nucleic acid- (LNA-) based anti-miRs, which have been shown to be more stable and less toxic in inhibiting endogenous miRNAs in vivo [77, 78]. Elmén et al. [78] used LNA-antimiR for efficient silencing of *miR-122* in primates, and a Phase 1 study is currently underway in humans. Another strategy recently developed to inhibit multiple miRNAs is called miR-sponge. miRNA sponges are transcripts that contain multiple tandem binding sites to specific miRNAs [79]. Sponge mRNA can be expressed from stably integrated transgenes in vivo to silence the target miRNAs and can perform comparable to antisense oligonucleotides. In case of miRs which are encoded in cluster, for example, *miR-17~92* family (and its paralogs, together encoding 15 miRNAs), it will be more efficient to generate a transgenic mouse with miR-cluster sponge to inhibit than deleting each miR individually [80].

On the other hand, restoration of tumor suppressor miRNAs can be achieved by synthetic miRNA mimics, which are usually double stranded and chemically modified (2′-O-methyl with phosphorothioate modifications). Use of *miR-15a* and *miR-29* mimics in prostate and AML cell lines, respectively, induced apoptosis [5, 81]. Another method to increase expression of tumor suppressor miRNAs is adenovirus-associated vectors (AAVs). AAV-mediated *miR-26* delivery into a murine model of liver cancer shows promise for miR replacement therapy [82]. AAV-based vectors do not integrate into the genome and are eliminated efficiently with minimal toxicity as seen in Phase I and Phase II clinical trials of about 200 patients [83, 84].

Other formulations like cationic lipids or liposomes, polymers, and nanoparticles have recently become popular to increase the efficiency of oligonucleotide uptake [5]. Liposomes consist of an aqueous compartment enclosed by a phospholipid bilayer and form stabilized complexes on electrostatic interactions with oligonucleotides [85]. In the studies so far, liposomes have been shown to induce hypersensitive reactions owing to toxicity, and hence research efforts to maximize their benefits are underway [86]. Polymers and nanoparticles on the other hand are promising because they provide improved delivery and stability with minimal in vivo toxicity [5, 87].

## 6. Future Directions

From the past and ongoing work on miRNAs in lymphomas, it is evident that deregulation of miRNA expression is one of the critical steps in pathogenesis of several lymphomas. Similar to mRNA, miRNA expression profiling can successfully classify various lymphomas into therapeutic outcome or survival categories. Identification of novel miRNA genes by new technologies like deep sequencing can further enhance the scope of miRNA functions and therapeutic applications [88]. In order to better understand the role of miRNAs in various lymphomas, future work should involve studying the targets involved in lymphomas and understand the mechanisms associated with them. In addition, generation of transgenic and knockout mouse models of lymphoma-associated miRNAs will help understand mechanisms underlying pathogenesis of these malignancies. Development of miRNA-based therapeutics faces similar challenges such as small interfering RNA therapeutics, potential off-target effects, safety, and mode of delivery (reviewed by Garzon et al. [5]). Hence, Mouse models of gain and loss of function of various miRNAs can be proved useful for respective miR-inhibitor or mimic-based therapies. These experiments can also lead to development of optimal formulation and delivery techniques.

## Figures and Tables

**Table 1:** miRs as associated with disease outcome.

	Poor prognosis	Favorable outcome	Reference
Non-Hodgkin lymphoma			

DLBCL	*miR-127*, *miR-222 *		[48, 51]
	*miR-637*, *miR-608*, *miR-302 *	*miR-330*, *miR-30e*, *miR-425*, *miR-27a*, *miR-24*, *miR-23a*, *miR-199b*, miR-199a*, *miR-100 *	[56]
BL	*miR-155*		
MCL	*miR-29*, *miR-20b *		[57, 58]

Hodgkin lymphoma			

HL	*miR-21*		[38]
TCL, SzS	*miR-125*		[59]

**Table 2:** Summary of up- and downregulated miRNA expression profiles in major types of lymphomas.

Lymphoma	Studies	Upregulated miRNAs	Downregulated miRNAs	Reference
Diffuse large B-cell lymphoma	*DLBCL* (*n* = 59*primary; n* = 27* cell lines) *	See reference	See reference	[49]
	*DLBCL (n* = 98*primary tumor;n* = 12* normal lymphocyte population)*	*hsa-miR-100*, *hsa-miR-10b*, *hsa-miR-125b*, *hsa-miR-143*, *hsa-miR-145*, *hsa-miR-155*, *hsa-miR-21*, *hsa-miR-34a*, *hsa-miR-451*, *hsa-miR-9 *	**hsa-miR-150**, *hsa-miR-181a*, *hsa-miR-189*, *hsa-miR-223*, *hsa-miR-361*, *hsa-miR-363*, *hsa-miR-495*, *hsa-miR-584*, *hsa-miR-625*, *hsa-miR-768-5p *	[56]
	*DLBCL (n* = 58*) versus LN (n* = 7)	*hsa-miR-210*, *hsa-miR-155*, *hsa-miR-106a*, *hsa-miR-17-5p *	**hsa-miR-150**, *hsa-miR-145*, *hsa-miR-328*, *hsa-miR-139*, *hsa-miR-99a*, *hsa-miR-10a*, *hsa-miR-95*, *hsa-miR-149*, *hsa-miR-320*, *hsa-miR-151*, *hsa-miR-let-7e *	[51]

Follicular lymphoma	*FL*(*n* = 46*) versus LN (n* = 7)	*hsa-miR-9*, hsa-miR-9*, *hsa-miR-301*, *hsa-miR-213*, *hsa-miR-330*, *hsa-miR-106a*, *hsa-miR-338*, *hsa-miR-155*, *hsa-miR-210 *	*hsa-miR-320*, *hsa-miR-149*, *hsa-miR-139 *	[51]

Mantle cell lymphoma	*MCL (n* = 30)	*hsa-miR-124a*, *hsa-miR-155*, *hsa-miR-328*, *hsa-miR-326*, *hsa-miR-302c*, *hsa-miR-345*, hsa-miR-373*, and *hsa-miR-210 *	*hsa-miR-29a/b/c*, *hsa-miR-142-3p/5p*, **hsa-miR-150**, and *hsa-miR-15a/b *	[54]

Burkitt lymphoma	*BL (n* = 10*) versus CLL*	*hsa-miR-130b*, *hsa-miR-154*, *hsa-miR-155*, *hsa-miR-29b*, *hsa-miR-29c*, *hsa-miR-637*, *hsa-miR-658*, *hsa-miR-193a-5p*, *hsa-miR-886-5p*, *hsa-miR-768-5p*, *hsa-miR-101*, *hsa-miR-933*, *hsa-miR-371-5p*, *hsa-miR-675*, *hsa-miR-150*, *hsa-miR-874*, *hsa-miR-181a*, *hsa-miR-30c*, *ebv-hsa-miR-BHRF1-2*, *hsa-miR-628-3p *		[58]
	*cMyc +* *BL* (*n* = 10)* versus* *cMyc − BL ( n* = 9)	*hsa-miR-17-5p*, *hsa-miR-20a *	hsa-miR-9*, *hsa-miR-34b *	[64]

Hodgkin lymphoma	*cHL (n* = 3*, cell lines)*	*hsa-miR-17~92*, *hsa-miR-16*, *hsa-miR-15*, *hsa-miR-21*, *hsa-miR-24* and *hsa-miR-155 *	**hsa-miR-150**	[39]
	*HL/HRS cell versus GC B cells*	*hsa-miR-20a*, *hsa-miR-21*, *hsa-miR-9*, *hsa-miR-155*, *hsa-miR-16*, *hsa-miR-140*, *hsa-miR-18a*, *hsa-miR-30b*, *hsa-miR-30a-5p*, *hsa-miR-196a*, *hsa-miR-374*, *hsa-miR-186 *	*hsa-miR-520a*, *hsa-miR-200a* and *hsa-miR-614 *	[36]
	*cHL (primary, n* = 49)	*hsa-miR-96*, *hsa-miR-128a*, *hsa-miR-128b *		[38]
	*HL cell lines (n* = 3*, L-428 & HD-MY-Z, L-1236)*	*hsa-miR-34a*, *hsa-miR-128b*, *hsa-miR-129*, *hsa-miR-200a *	*hsa-miR-122a*, *hsa-miR-154*, *hsa-miR-302d*, *hsa-miR-371 *	[38]
	*cHL cell lines*	*hsa-miR-21*, *hsa-miR-27a*, *hsa-miR-147*, *hsa-miR-182*, *hsa-miR-183*, *hsa-miR-216 *	*hsa-miR-126*, *hsa-miR-135a*, *hsa-miR-204 *	[38]
	*EBV + versus EBV − cHL*	*hsa-miR-28*, *hsa-miR-130b*, *hsa-miR-132*, *hsa-miR-140*, *hsa-miR-330 *	*hsa-miR-96*, *hsa-miR-128a*, *hsa-miR-128b*, *hsa-miR-129*, *hsa-miR-205 *	[38]

T-cell lymphoma	*Sezary syndrome/SzS (n* = 17)	*hsa-miR-145*, *hsa-miR-574-5p*, *hsa-miR-200c*, hsa-miR-199a*, *hsa-miR-143*, *hsa-miR-214*, *hsa-miR-98*, *hsa-miR-518a-3p*, *hsa-miR-7 *	*hsa-miR-342*, *hsa-miR-223*, *hsa-miR-150*, hsa-miR-189(24*), *hsa-miR-186*, *hsa-miR-423-3p*, *hsa-miR-92*, *hsa-miR-181a*, *hsa-miR-191*, *hsa-miR-376a *	[59]
	*Lymphocyte variant Hypereosinophilic syndrome/L-HES (n* = 7)	Let-7b, *hsa-miR-221*, *hsa-miR-222*,	*hsa-miR-26a*, *hsa-miR-31*, *hsa-miR-95*, *hsa-miR-99a*, *hsa-miR-100*, *hsa-miR-126*, *hsa-miR-130a*, *hsa-miR-135b*, *hsa-miR-135a*, *hsa-miR-151*, *hsa-miR-181a*, *hsa-miR-181b*, *hsa-miR-193a*, *hsa-miR-213*, *hsa-miR-215*, *hsa-miR-335*, *hsa-miR-340*, *hsa-miR-125a *	[65]

## References

[1] Lee Y, Jeon K, Lee JT, Kim S, Kim VN (2002). MicroRNA maturation: stepwise processing and subcellular localization. *EMBO Journal*.

[2] Lee Y, Ahn C, Han J (2003). The nuclear RNase III Drosha initiates microRNA processing. *Nature*.

[3] Gregory RI, Chendrimada TP, Cooch N, Shiekhattar R (2005). Human RISC couples microRNA biogenesis and posttranscriptional gene silencing. *Cell*.

[4] Doench JG, Sharp PA (2004). Specificity of microRNA target selection in translational repression. *Genes and Development*.

[5] Garzon R, Marcucci G, Croce CM (2010). Targeting microRNAs in cancer: rationale, strategies and challenges. *Nature Reviews Drug Discovery*.

[6] Rodriguez A, Griffiths-Jones S, Ashurst JL, Bradley A (2004). Identification of mammalian microRNA host genes and transcription units. *Genome Research*.

[7] Sevignani C, Calin GA, Nnadi SC (2007). MicroRNA genes are frequently located near mouse cancer susceptibility loci. *Proceedings of the National Academy of Sciences of the United States of America*.

[8] Saito Y, Liang G, Egger G (2006). Specific activation of microRNA-127 with downregulation of the proto-oncogene BCL6 by chromatin-modifying drugs in human cancer cells. *Cancer Cell*.

[9] Lujambio A, Ropero S, Ballestar E (2007). Genetic unmasking of an epigenetically silenced microRNA in human cancer cells. *Cancer Research*.

[10] Hackanson B, Bennett KL, Brena RM (2008). Epigenetic modification of CCAAT/enhancer binding protein *α* expression in acute myeloid leukemia. *Cancer Research*.

[11] Kumar MS, Pester RE, Chen CY (2009). Dicer1 functions as a haploinsufficient tumor suppressor. *Genes and Development*.

[12] He L, He X, Lim LP (2007). A microRNA component of the p53 tumour suppressor network. *Nature*.

[13] Chang TC, Wentzel EA, Kent OA (2007). Transactivation of miR-34a by p53 broadlyinfluences gene expression andpromotesapoptosis. *Molecular Cell*.

[14] Tam W, Ben-Yehuda D, Hayward WS (1997). bic, a novel gene activated by provital insertions in avian leukosis virus-induced lymphomas, is likely to function through its noncoding RNA. *Molecular and Cellular Biology*.

[15] Eis PS, Tam W, Sun L (2005). Accumulation of miR-155 and BIC RNA in human B cell lymphomas. *Proceedings of the National Academy of Sciences of the United States of America*.

[16] Rai D, Karanti S, Jung I, Dahia PLM, Aguiar RCT (2008). Coordinated expression of microRNA-155 and predicted target genes in diffuse large B-cell lymphoma. *Cancer Genetics and Cytogenetics*.

[17] Kluiver J, Poppema S, de Jong D (2005). BIC and miR-155 are highly expressed in Hodgkin, primary mediastinal and diffuse large B cell lymphomas. *Journal of Pathology*.

[18] Calin GA, Ferracin M, Cimmino A (2005). A microRNA signature associated with prognosis and progression in chronic lymphocytic leukemia. *The New England Journal of Medicine*.

[19] Costinean S, Zanesi N, Pekarsky Y (2006). Pre-B cell proliferation and lymphoblastic leukemia/high-grade lymphoma in E*μ*-miR155 transgenic mice. *Proceedings of the National Academy of Sciences of the United States of America*.

[20] Gottwein E, Mukherjee N, Sachse C (2007). A viral microRNA functions as an orthologue of cellular miR-155. *Nature*.

[21] Zhao Y, Yao Y, Xu H (2009). A functional microRNA-155 ortholog encoded by the oncogenic Marek’s disease virus. *Journal of Virology*.

[22] Costinean S, Sandhu SK, Pedersen IM (2009). Src homology 2 domain-containing inositol-5-phosphatase and CCAAT enhancer-binding protein *β* are targeted by miR-155 in B cells of E*μ*-MiR-155 transgenic mice. *Blood*.

[23] O’Connell RM, Chaudhuri AA, Rao DS, Baltimore D (2009). Inositol phosphatase SHIP1 is a primary target of miR-155. *Proceedings of the National Academy of Sciences of the United States of America*.

[24] Rodriguez A, Vigorito E, Clare S (2007). Requirement of bic/microRNA-155 for normal immune function. *Science*.

[25] Thai TOH, Calado DP, Casola S (2007). Regulation of the germinal center response by MicroRNA-155. *Science*.

[26] Yin Q, Wang X, McBride J, Fewell C, Flemington E (2008). B-cell receptor activation induces BIC/miR-155 expression through a conserved AP-1 element. *Journal of Biological Chemistry*.

[27] Ota A, Tagawa H, Karnan S (2004). Identification and characterization of a novel gene, C13orf25, as a target for 13q31-q32 amplification in malignant lymphoma. *Cancer Research*.

[28] He L, Thomson JM, Hemann MT (2005). A microRNA polycistron as a potential human oncogene. *Nature*.

[29] Ventura A, Young AG, Winslow MM (2008). Targeted Deletion Reveals Essential and Overlapping Functions of the miR-17*∼*92 Family of miRNA Clusters. *Cell*.

[30] O’Donnell KA, Wentzel EA, Zeller KI, Dang CV, Mendell JT (2005). c-Myc-regulated microRNAs modulate E2F1 expression. *Nature*.

[31] Xiao C, Srinivasan L, Calado DP (2008). Lymphoproliferative disease and autoimmunity in mice with increased miR-17-92 expression in lymphocytes. *Nature Immunology*.

[32] Mu P, Han YC, Betel D (2009). Genetic dissection of the miR-17∼92 cluster of microRNAs in Myc-induced B-cell lymphomas. *Genes and Development*.

[33] Olive V, Bennett MJ, Walker JC (2009). miR-19 is a key oncogenic component of mir-17-92. *Genes and Development*.

[34] Dews M, Fox JL, Hultine S (2010). The Myc-miR-17∼92 axis blunts TGF*β* signaling and production of multiple TGF*β*-dependent antiangiogenic factors. *Cancer Research*.

[35] Küppers R (2009). Molecular biology of Hodgkin lymphoma. *Hematology / American Society of Hematology. Education Program*.

[36] Van Vlierberghe P, De Weer AN, Mestdagh P (2009). Comparison of miRNA profiles of microdissected Hodgkin/Reed-Sternberg cells and Hodgkin cell lines versus CD77+ B-cells reveals a distinct subset of differentially expressed miRNAs. *British Journal of Haematology*.

[37] Nie K, Gomez M, Landgraf P (2008). MicroRNA-mediated down-regulation of PRDM1/Blimp-1 in Hodgkin/Reed- Sternberg cells: a potential pathogenetic lesion in Hodgkin lymphomas. *American Journal of Pathology*.

[38] Navarro A, Gaya A, Martinez A (2008). MicroRNA expression profiling in classic Hodgkin lymphoma. *Blood*.

[39] Gibcus JH, Tan LUP, Harms G (2009). Hodgkin lymphoma cell lines are characterized by a specific miRNA expression profile. *Neoplasia*.

[40] Mancao C, Altmann M, Jungnickel B, Hammerschmidt W (2005). Rescue of "crippled" germinal center B cells from apoptosis by Epstein-Barr virus. *Blood*.

[41] Gatto G, Rossi A, Rossi D, Kroening S, Bonatti S, Mallardo M (2008). Epstein-Barr virus latent membrane protein 1 trans-activates miR-155 transcription through the NF-*κ*B pathway. *Nucleic Acids Research*.

[42] Westin JR, Fayad LE (2009). Beyond R-CHOP and the IPI in large-cell lymphoma: molecular markers as an opportunity for stratification. *Current Hematologic Malignancy Reports*.

[43] Rosenwald A, Wright G, Chan WC (2002). The use of molecular profiling to predict survival after chemotherapy for diffuse large-B-cell lymphoma. *The New England Journal of Medicine*.

[44] Hans CP, Weisenburger DD, Greiner TC (2004). Confirmation of the molecular classification of diffuse large B-cell lymphoma by immunohistochemistry using a tissue microarray. *Blood*.

[45] Jaffe ES, Harris NL, Stein H, Isaacson PG (2008). Classification of lymphoid neoplasms: the microscope as a tool for disease discovery. *Blood*.

[46] Alizadeh AA, Elsen MB, Davis RE (2000). Distinct types of diffuse large B-cell lymphoma identified by gene expression profiling. *Nature*.

[47] Lenz G, Staudt LM (2010). Aggressive lymphomas. *The New England Journal of Medicine*.

[48] Malumbres R, Sarosiek KA, Cubedo E (2009). Differentiation stage-specific expression of microRNAs in B lymphocytes and diffuse large B-cell lymphomas. *Blood*.

[49] Li C, Kim SW, Rai D (2009). Copy number abnormalities, MYC activity, and the genetic fingerprint of normal B cells mechanistically define the microRNA profile of diffuse large B-cell lymphoma. *Blood*.

[50] Calin GA, Dumitru CD, Shimizu M (2002). Frequent deletions and down-regulation of micro-RNA genes miR15 and miR16 at 13q14 in chronic lymphocytic leukemia. *Proceedings of the National Academy of Sciences of the United States of America*.

[51] Roehle A, Hoefig KP, Repsilber D (2008). MicroRNA signatures characterize diffuse large B-cell lymphomas and follicular lymphomas. *British Journal of Haematology*.

[52] Rai D, Kim SW, McKeller MR, Dahia PLM, Aguiar RCT (2010). Targeting of SMAD5 links microRNA-155 to the TGF-*β* pathway and lymphomagenesis. *Proceedings of the National Academy of Sciences of the United States of America*.

[53] Scandurra M, Rossi D, Deambrogi C (2010). Genomic profiling of Richter’s syndrome: recurrent lesions and differences with de novo diffuse large B-cell lymphomas. *Hematological Oncology*.

[54] Zhao JJ, Lin J, Lwin T (2010). MicroRNA expression profile and identification of miR-29 as a prognostic marker and pathogenetic factor by targeting CDK6 in mantle cell lymphoma. *Blood*.

[55] Pekarsky Y, Santanam U, Cimmino A (2006). Tcl1 expression in chronic lymphocytic leukemia is regulated by miR-29 and miR-181. *Cancer Research*.

[56] Lawrie CH, Chi J, Taylor S (2009). Expression of microRNAs in diffuse large B cell lymphoma is associated with immunophenotype, survival and transformation from follicular lymphoma. *Journal of Cellular and Molecular Medicine*.

[57] Di L, Gómez-López G, Sánchez-Beato M (2010). Mantle cell lymphoma: transcriptional regulation by microRNAs. *Leukemia*.

[58] Zhang J, Jima DD, Jacobs C (2009). Patterns of microRNA expression characterize stages of human B-cell differentiation. *Blood*.

[59] Ballabio E, Mitchell T, Van Kester MS (2010). MicroRNA expression in Sézary syndrome: identification, function, and diagnostic potential. *Blood*.

[60] Ivanovska I, Ball AS, Diaz RL (2008). MicroRNAs in the miR-106b family regulate p21/CDKN1A and promote cell cycle progression. *Molecular and Cellular Biology*.

[61] Jaffe ES (2009). The 2008 WHO classification of lymphomas: implications for clinical practice and translational research. *Hematology / American Society of Hematology. Education Program*.

[62] Carbone A, Gloghini A, Aiello A, Testi A, Cabras A (2010). B-cell lymphomas with features intermediate between distinct pathologic entities. From pathogenesis to pathology. *Human Pathology*.

[63] De Falco G, Antonicelli G, Onnis A, Lazzi S, Bellan C, Leoncini L (2009). Role of EBV in microRNA dysregulation in Burkitt lymphoma. *Seminars in Cancer Biology*.

[64] Onnis A, de Falco G, Antonicelli G (2010). Alteration of microRNAs regulated by c-Myc in Burkitt Lymphoma. *PLoS ONE*.

[65] Ravoet M, Sibille C, Gu C (2009). Molecular profiling of CD3CD4 T cells from patients with the lymphocytic variant of hypereosinophilic syndrome reveals targeting of growth control pathways. *Blood*.

[66] Beck-Engeser GB, Lum AM, Huppi K, Caplen NJ, Wang BB, Wabl M (2008). Pvt1-encoded microRNAs in oncogenesis. *Retrovirology*.

[67] Robertus JL, Kluiver J, Weggemans C (2010). MiRNA profiling in B non-Hodgkin lymphoma: a MYC-related miRNA profile characterizes Burkitt lymphoma. *British Journal of Haematology*.

[68] Akao Y, Nakagawa Y, Kitade Y, Kinoshita T, Naoe T (2007). Downregulation of microRNAs-143 and -145 in B-cell malignancies. *Cancer Science*.

[69] Ott G, Rosenwald A (2008). Molecular pathogenesis of follicular lymphoma. *Haematologica*.

[70] Tili E, Michaille JJ, Cimino A (2007). Modulation of miR-155 and miR-125b levels following lipopolysaccharide/TNF- *α* stimulation and their possible roles in regulating the response to endotoxin shock. *Journal of Immunology*.

[71] Lawrie CH, Gal S, Dunlop HM (2008). Detection of elevated levels of tumour-associated microRNAs in serum of patients with diffuse large B-cell lymphoma. *British Journal of Haematology*.

[72] Mitchell PS, Parkin RK, Kroh EM (2008). Circulating microRNAs as stable blood-based markers for cancer detection. *Proceedings of the National Academy of Sciences of the United States of America*.

[73] Xiao C, Calado DP, Galler G (2007). MiR-150 controls B cell differentiation by targeting the transcription factor c-Myb. *Cell*.

[74] Trang P, Medina PP, Wiggins JF (2010). Regression of murine lung tumors by the let-7 microRNA. *Oncogene*.

[75] Chan JA, Krichevsky AM, Kosik KS (2005). MicroRNA-21 is an antiapoptotic factor in human glioblastoma cells. *Cancer Research*.

[76] Si ML, Zhu S, Wu H, Lu Z, Wu F, Mo YY (2007). miR-21-mediated tumor growth. *Oncogene*.

[77] Vester B, Wengel J (2004). LNA (Locked nucleic acid): high-affinity targeting of complementary RNA and DNA. *Biochemistry*.

[78] Elmén J, Lindow M, Schütz S (2008). LNA-mediated microRNA silencing in non-human primates. *Nature*.

[79] Ebert MS, Neilson JR, Sharp PA (2007). MicroRNA sponges: competitive inhibitors of small RNAs in mammalian cells. *Nature Methods*.

[80] Hammond SM (2007). Soaking up small RNAs. *Nature Methods*.

[81] Bonci D, Coppola V, Musumeci M (2008). The miR-15a-miR-16-1 cluster controls prostate cancer by targeting multiple oncogenic activities. *Nature Medicine*.

[82] Kota J, Chivukula RR, O’Donnell KA (2009). Therapeutic microRNA delivery suppresses tumorigenesis in a murine liver cancer model. *Cell*.

[83] Michelfelder S, Trepel M (2009). Adeno-Associated Viral Vectors and Their Redirection to Cell-Type Specific Receptors. *Advances in Genetics*.

[84] Aagaard L, Rossi JJ (2007). RNAi therapeutics: principles, prospects and challenges. *Advanced Drug Delivery Reviews*.

[85] Zhao X, Pan F, Holt CM, Lewis AL, Lu JR (2009). Controlled delivery of antisense oligonucleotides: a brief review of current strategies. *Expert Opinion on Drug Delivery*.

[86] Zuhorn IS, Engberts JBFN, Hoekstra D (2007). Gene delivery by cationic lipid vectors: overcoming cellular barriers. *European Biophysics Journal*.

[87] Chirila TV, Rakoczy PE, Garrett KL, Lou X, Constable IJ (2002). The use of synthetic polymers for delivery of therapeutic antisense oligodeoxynucleotides. *Biomaterials*.

[88] Jima DD, Zhang J, Jacobs C (2010). Deep sequencing of the small RNA transcriptome of normal and malignant human B cells identifies hundreds of novel microRNAs. *Blood*.

